# Quantitative Damage Detection and Evolution in Composite Structures Using Digital Image Correlation, Machine Learning, and Peridynamics

**DOI:** 10.3390/ma19101917

**Published:** 2026-05-07

**Authors:** Tomas Vaitkūnas, Elena Jasiūnienė, Justas Griškevičius, Vykintas Samaitis, Paulius Griškevičius

**Affiliations:** 1 Department of Mechanical Engineering, Kaunas University of Technology, Studentu Str. 56, LT-51424 Kaunas, Lithuania; 2 Department of Electronics Engineering, Kaunas University of Technology, Studentu Str. 50, LT-51368 Kaunas, Lithuania; elena.jasiuniene@ktu.lt; 3 Prof. K. Barsauskas Ultrasound Research Institute, Kaunas University of Technology, K.Baršausko St. 59, LT-51423 Kaunas, Lithuania; vykintas.samaitis@ktu.lt; 4 Department of Applied Informatics, Kaunas University of Technology, Studentu str.50, LT-51368 Kaunas, Lithuania

**Keywords:** structural health monitoring, digital image correlation, machine learning, peridynamics, composite materials, damage detection, damage evolution, fatigue, inverse identification

## Abstract

Structural health monitoring (SHM) of composite structures using surface strain fields measured by digital image correlation (DIC) has been widely demonstrated; however, accurate damage quantification remains challenging. This study proposes a hybrid framework integrating finite element (FE) modeling, machine learning (ML), and peridynamics (PD). A CFRP specimen with a notch was subjected to cyclic loading, and damage evolution was monitored using DIC and validated by ultrasound measurements. A validated FE model generated synthetic strain-field datasets for ML training, enabling defect detection and quantitative characterization directly from surface strains. The trained models achieved high accuracy, including perfect notch detection and low prediction errors. A calibrated PD model captured internal damage evolution and fatigue behavior. The combined DIC–ML–PD approach enables accurate, non-contact damage identification and prognosis, supporting physics-informed digital twins for composite structures.

## 1. Introduction

Various composite materials, due to the possibility of gaining better material performance, have been developed and used since the start of the XX century. This especially applies to carbon and glass fiber reinforced polymer composites (CFRP and GFRP), which have high strength–low mass properties, corrosion and fatigue resistance, making them a necessary and irreplaceable material for wind energy, aviation, sport, and other industries [1]. On the other hand, a complex multi-material structure requires different manufacturing operations, which are prone to inducing material defects. Defects can also be induced during structure operation and are the leading cause of early structure failure [2]. Because of this, structural health monitoring (SHM) of composite structures becomes very important and a challenging task. Different non-destructive testing methods have already been developed (e.g., ultrasound [3], X-ray tomography [4], acoustic emission [5], other [6]), although requiring contact with the tested structure and lab equipment (applies for X-ray tomography) limits composite SHM capabilities.

To overcome these limitations, recent research has explored the integration of carbon nanotube (CNT)-based piezoresistive sensors for in situ monitoring [7], particularly in aerospace and wind energy components. While these nanotechnology-based sensors offer high sensitivity to strain (and can even compensate for temperature change effects [8]) and can be embedded directly within the composite laminate, they also suffer from drawbacks, including signal hysteresis, especially at cyclic loadings, baseline drift due to other environmental factors like humidity, and the technical difficulty of ensuring uniform CNT dispersion in large-scale structures. Other emerging methodologies, such as Terahertz (THz) imaging [9] and Infrared Thermography (IRT) [10], have been proposed as non-contact alternatives for identifying subsurface defects. Although effective for detecting delamination, THz imaging remains costly and limited by its penetration depth in highly conductive carbon-fiber composites, while IRT is highly dependent on thermal excitation uniformity and often lacks the spatial resolution required for precise strain localization. Consequently, there is a growing need for a versatile, cost-effective, and full-field monitoring solution that can operate under diverse conditions without compromising the structural integrity of the composite.

Digital image correlation (DIC), as a full-field optical measurement technique, was created by Sutton in 1980 [11]. The object surface points (a speckle pattern on the object surface is necessary) are tracked by optical cameras, and after that, the images are postprocessed in software to compute surface displacements and strains. Rather than tracking separate points, subsets of points are tracked and used to compute surface point displacements and strains. A setup consisting of two cameras is capable of measuring out-of-plane surface displacements (3D DIC). There have already been studies reporting successful results when identifying damages in composite structures from unevenness in DIC-measured surface strain fields [12]. However, it is still difficult to identify the amount of damage in the structure. Jasiuniene et al. [13] focused on different locations and sizes of damage detectability by DIC, but much attention was not paid to determining the amount of the damage. Recent development of AI enables the employment of an AI for predicting damage directly from DIC images [14]. There have already been proposed studies [15,16,17,18,19] where a database of FEM models is used to learn AI, and then the amount of damage can be evaluated.

Nevertheless, the conventional FEM issue in the case of discontinuity is that the FEM does not converge. Additional methods (initial cracks, special elements at the crack zone, etc. [20]), which rely on multiple parameters difficult to establish, are necessary. The problem resulted from the FE formulation in terms of spatial derivatives, which are not valid for discontinuities. This becomes important when modeling fracture problems, such as crack growth and/or fatigue failure. In order to simulate continuous and discontinuous fields without any special methods, reformulation of classical continuum mechanics (CCM)—non-local integral peridynamics (PD) theory, was originally proposed by Silling in 2000 [21]. PD theory does not face problems modeling any cracks, even very complex ones, such as crack branching [17]. PD is already applied to isotropic and composite materials [22], especially (dynamic) cracking [19], cyclic damage and failure [23], even in composites. The first proposed by Silling PD theory [21] was bond-based peridynamics (BBPD), and it resulted in a fixed Poisson’s ratio of material value of 0.25 for the 3D model and 0.33 for the 2D plane stress case. Later, free of such limitations, state-based peridynamics (SBPD) was created [24], although it was formulated in terms of new mathematical objects—states, which require special software rather than FEM. Madenci [25] suggested BBPD with a bond rotation model for a one-layer composite. The model is both free of a fixed Poisson’s ratio value and can even be constructed from existing truss finite elements. In general, PD theory-related publications have been increasing exponentially since 2000 [26].

While digital image correlation (DIC) has been extensively utilized for identifying damage in composite structures, the method remains limited to qualitative damage detection from surface strain fields. Significant gaps exist in quantifying the precise extent of damage and, more critically, in predicting the remaining fatigue life based on DIC measurements. To the best of the authors’ knowledge, a synergistic approach that bridges optical surface measurements with AI-driven quantification and advanced PD-based life-cycle prognosis has not yet been reported. The novelty of this study lies in the integration of three distinct methodologies into a unified diagnostic and prognostic framework: (1) high-resolution DIC for real-time surface strain monitoring, (2) artificial intelligence (AI) for the automated identification of defect geometry and spatial positioning, and (3) peridynamics (PD) theory for modeling crack propagation. Specifically, this study extends the bond-based peridynamics (BBPD) model with bond rotation originally proposed by Madenci [25] for single laminates to multilayered composite structures, enabling accurate damage growth prediction. The proposed methodology involves cyclic loading tests on notched CFRP specimens, where DIC-measured surface strains serve as the primary data source. These measurements are processed by an AI model, trained on validated FE simulations, to quantify the geometry and location of defects. The identified damage state then informs the enhanced BBPD model to simulate crack propagation and forecast the remaining cycles to failure. Finally, ultrasonic testing is employed to validate the AI-driven diagnostics. This approach establishes a comprehensive, non-contact SHM system capable of evaluating and forecasting structural integrity and lifetime solely from surface-level optical data.

## 2. Materials and Methods

### 2.1. Specimens

Commercially available carbon fiber reinforced polymer (CFRP) composite plate OKE with dimensions of 350 × 150 × 2 mm was used for preparing a specimen with damage. The OKE plate manufacturing process utilizes high-pressure thermal consolidation (press-molding) of HT-3-K carbon prepregs with a transparent epoxy resin system. The plate material properties, given by the manufacturer [27], are: fiber volume fracture of 60–65%, density of 1500–1600 kg/m^3^, effective elastic modulus of 50–70 GPa, ultimate strength of 600–775 MPa.

**Figure 1:**
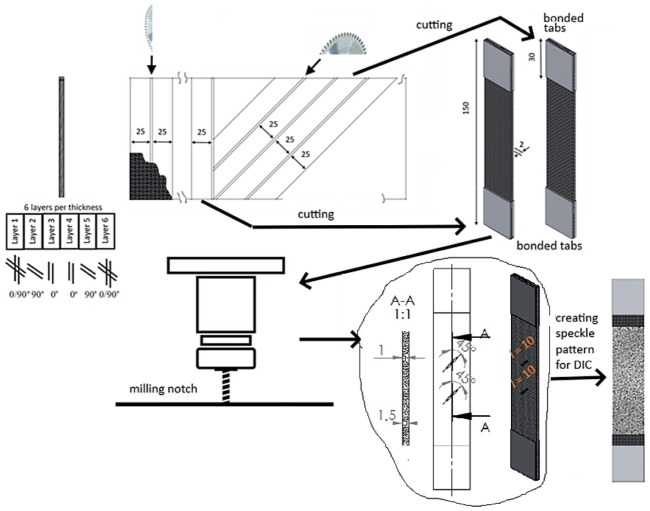
CFRP specimens for tensile and cyclic tests.

The plate has 6 layers in total with a layup of [(0/90)°/90°/0°]s as shown in Figure 1. Firstly, tensile specimens (Figure 1) were prepared from the plate to find the material’s effective properties and calibrate numerical models. Specimens were cut by a circular table saw at directions of 0° and 45° from the CFRP plate, as shown in Figure 1, resulting in fiber orientation angles [(0/90)°/90°/0°]s and [(±45)°/−45°/45°]s. Tensile specimens’ dimensions were taken according to the standard ASTM D3039 [28]: width of 25 mm; while there is no direct requirement for specimen length, it was taken to be the same as the plate dimension—150 mm. Tabs of dimensions of 25 × 30 mm, made of 1.5 mm thick aluminum sheet, were bonded to both specimen ends of both sides to prevent failure at the machine grips. Due to the high quality and the inherent homogeneity of the high-pressure hot-pressed OKE laminate, a sample size of three tensile specimens was deemed sufficient for each selected layup.

Specimen for damage monitoring was prepared from the same tensile specimens with layup of [(0/90)°/90°/0°]s (see Figure 1) by milling 2 notches of the same width of 1 mm, length of 10 mm, 45° orientation angle to tensile direction and depths of 1 mm (upper notch) and 1.5 mm (lower notch). The distance between the notches was selected to be 21 mm to prevent their interaction during cyclic damage growth. A 2-flute carbide end mill with CVD coating was used. Mill diameter was equal to the notch width of 1 mm, and the mill corner radius was about 0.1 mm. As the primary objective was to observe the qualitative evolution of damage rather than to determine statistical fatigue properties (S-N curves), a single-sample approach was deemed appropriate for this phenomenological study.

### 2.2. Experimental Testing

#### 2.2.1. Tensile and Cyclic Tests with Digital Image Correlation

Tensile tests of the CFRP samples with layups of [(0/90)°/90°/0°]s and [(±45)°/−45°/45°]s were done on an electromechanical tensile testing machine (Instron 8862, Norwood, MA, USA) with a maximum force of 100 kN. Strains were measured by using an external extensometer. Force measurements were performed by the machine force cell. Tensile speed was 1 mm/min. Only specimens with effective tensile curves were taken from the test and used for PD model calibration.

**Figure 2:**
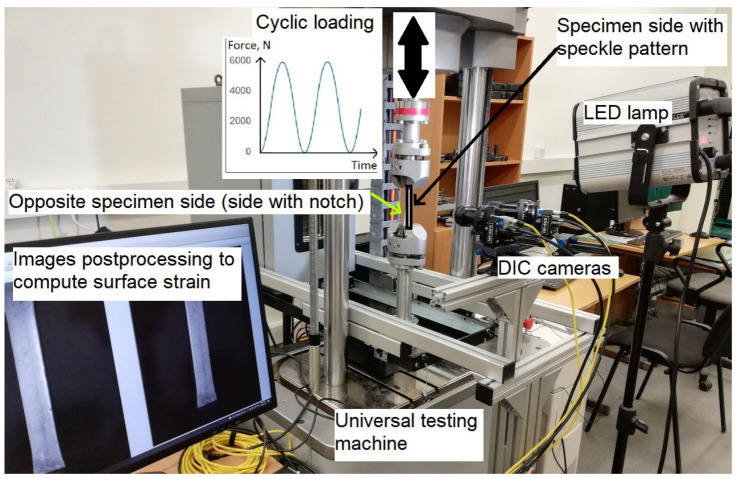
Cyclic testing with DIC experimental setup.

Cyclic test of the specimen with notches was run on a universal testing machine (ElectroPuls E10000, Instron, Norwood, MA, USA) (Figure 2) with a force up to 10 kN. Asymmetric (zero-based) sinus wave tensile cyclic loading (*R* = 0) with a maximum force of 6 kN and frequency of 5 Hz was used for the cyclic testing of the specimen. The selection of the asymmetric cyclic loading was based on low specimen thickness, which could result in buckling failure due to high compression forces at symmetric cyclic loading. Maximum force was selected to be 15% of the specimen failure load in order to prevent static damage in each loading cycle. The specimen side, opposite to the milled notch, was painted with white sprayed paint and coated with a speckled pattern necessary for DIC measurements, as shown in Figure 1. The pattern was created by spraying black paint using an airbrush, and the average pattern speckle size was 0.05 mm. Two DIC cameras acA4112-20 um (Basler AG, Ahrensburg, Germany) were used to capture the image of the specimen surface during the different cycles at the tensile loading peak. A 1000 W maximum power LED lamp Hedler Profilux LED 1000) was used for creating proper lighting for DIC measurements. Before the experiment, the DIC system was calibrated from 26 images of a 3 mm (9 × 9 points) calibration plate. The software VIC-3D-10.0.90 was used for image postprocessing. The calibration score was 0.047 < 0.1, which indicates proper calibration. The DIC parameters, used for image postprocessing in VIC-3D-10.0.90 software, are summarized in Table 1. Their resultant measurement uncertainty interval was 0.02 px.

#### 2.2.2. Ultrasonic Inspection

**Figure 3:**
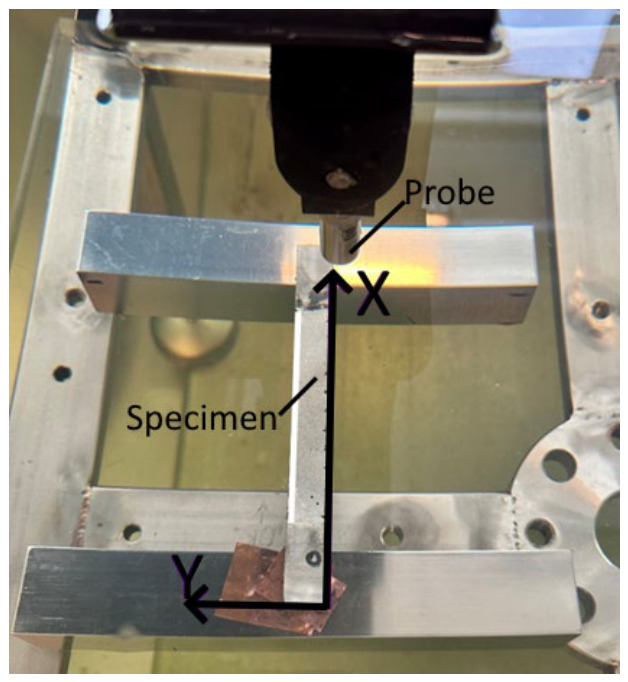
Ultrasound inspection set-up of the specimen with notches.

Ultrasound inspection setup consisted of a 10 MHz focused probe with a 2″ focal distance, focused on the surface of the specimen as shown in Figure 3. The excitation voltage was selected to be 100 V with a gain of 10 dB and a sampling frequency of 125 MHz using a 14-bit ADC, ensuring sufficient temporal resolution for accurate time-of-flight and depth estimation. Data acquisition was performed with an ADC delay of 60 µs and an acquisition window of 10 µs. No additional time-varying gain (TVG) or adaptive gain control was used. Measurements were performed in a pulse-echo mode. The scanning of the specimen with notches was performed after the cyclic tests and was carried out along the y-axis with increments along the x-axis. The scanning step was 0.1 mm. The scanning area had 1023 × 216 measurement points.

The acquired A-scan signals were analyzed in the time domain. No digital filtering was applied after the acquisition. C-scan images were generated by extracting the signal amplitude within selected time gates corresponding to specific depths below the surface, while B-scans were constructed from stacked A-scans and helped to visualize delamination depth.

The delamination detection criteria were based on localized amplitude increases and shadowing effects in the A-scan signals, occurring at consistent time-of-flight positions across neighboring scan points. This indicates the presence of an interface, which causes partial reflection and attenuation of the back-wall echo. Delamination depth can be estimated from the time-of-flight of the signal relative to the surface echo, while delamination area can be quantified from thresholded C-scan maps at the corresponding depth.

### 2.3. Peridynamics Theory

**Figure 4:**
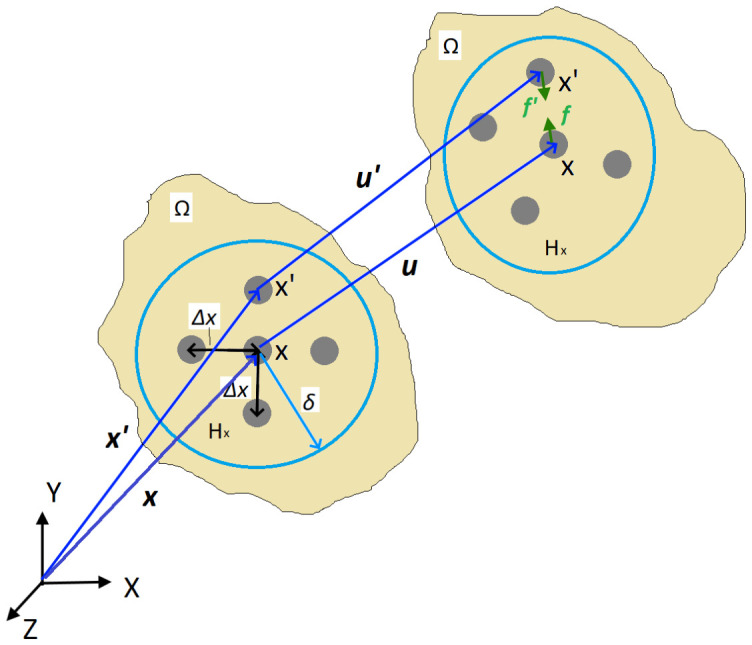
The original Silling proposed BBPD schematics.

PD theory reformulates the CCM, defining interactions between the equally spaced PD points *x* in the body domain *Ω* (Figure 4). An interaction between the PD points *x* and *x’* is called the PD bond $\xi_{xx^{\prime }}=x^{\prime }-x$ and is defined in the interaction range, known as PD horizon *H_x_*. Here, ***x*** and ***x’*** refer to points *x* and *x’* position vectors (Figure 4). For a conventional 3D PD model, PD points are spaced in a regular grid with the distance between the points *Δx* and the interaction range of the sphere with radius *δ*. After the deformation, PD bonds experience a stretch resulting in PD forces. The original Silling PD theory [21] assumed the PD bond $\xi_{xx^{\prime }}$ forces at points *x* and *x’* are equal in magnitude and parallel in direction, resulting in the BBPD formulation shown in Figure 4.

Then, the BBPD equation of motion is written [22]:

(1) $$\rho \ddot{u}\left(x,t\right)=\int_{H_{x}}f\left(u^{\prime }(x,t)-u(x,t),x^{\prime }-x,x\right)dV_{x}+b\left(x,t\right)$$

where ρ is the material density, ***u*** and ***u’*** are the displacement vectors of points *x* and *x’*, *t* is the time, ***f*** is the BBPD bond force vectors, *V_x_* is the volume of PD point *x*, and ***b***(***x***, t) is the body force vector. Optimum PD horizon *H_x_* size, based on PD convergence studies [29], is *δ* ≈ *3Δx*.

Stretch of each PD bond $\xi_{xx^{\prime }}$ after the deformation can be found from the PD points’ relative position $\xi_{xx^{\prime }}$ and relative displacement $\eta_{xx^{\prime }}=u^{\prime }-u$ vectors. Using notification y=x+u and $y^{\prime }=x^{\prime }+u^{\prime }$, the stretch can be expressed as:

(2) $$s_{xx^{\prime }}=\frac{\left(\left|y^{\prime }-y\right|-\left|\xi_{xx^{\prime }}\right|\right)}{\left|\xi_{xx^{\prime }}\right|}$$

The BBPD forces of the current PD bond $\xi_{xx^{\prime }}$ are computed from the stretch and PD bond stiffness as:

(3) $$f_{xx^{\prime }}=cs_{xx^{\prime }}\frac{y^{\prime }-y}{\left|y^{\prime }-y\right|}$$

where *c* is the BBPD bond stiffness. Critical stretch value *s_c_* is used to induce static failure in the PD model. Any PD bond is considered permanently failed and is deleted once the *s_xx’_* exceeds the *s_c_*, resulting in *c_xx’_* = 0 and then ***f_xx’_*** = 0. BBPD bond stiffness is derived by comparing the CCM and PD strain energy densities at the same deformation and depends on the elastic modulus of the material, PD horizon size and PD model formulation, as expressed by the following equation:

(4) $$c=\left\{\begin{array}{c} \frac{12E}{\pi \delta^{4}}\;3D\;\mathrm{case}\\ \frac{9E}{\pi \delta^{3}}\;2D\;\mathrm{plane}\;\mathrm{stress}\;\mathrm{case}\\ \frac{8E}{\pi \delta^{3}}\;2D\;\mathrm{plane}\;\mathrm{strain}\;\mathrm{case} \end{array}\right.$$

It is worth noticing that both PD model parameters, bond stiffness *c* and failure parameter critical PD bond stretch *s_c_*, are dependent on the PD horizon size and cannot be equalized to any CCM material constants and strength parameters.

#### 2.3.1. Bond-Based Peridynamics with Bond Rotation for Composite

**Figure 5:**
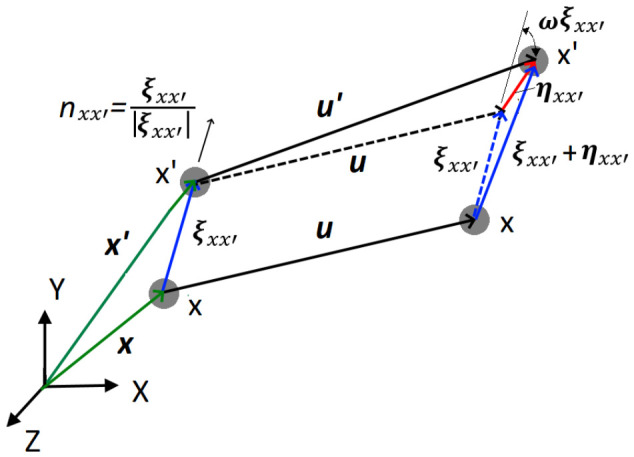
BBPD with PD bond rotation kinematics.

Because the BBPD model is described by only one material constant *c*, which depends on material elastic modulus *E* (Equation (4)), this results in a fixed material Poisson’s ratio of 0.25 for 3D and 2D plane strain, and 0.33 for 2D plain stress [30]. In order to circumvent this limitation, the SBPD theory, which uses additional mathematical objects–states, was developed [22]. The SBPD model cannot be constructed using existing FE, as opposed to BBPD, which can be implemented into FEM using truss elements as the PD bonds [31]. The possibility of creating a PD model without special software or programming skills is attractive for companies or other non-research institutions. Moreover, coupling between FEM and BBPD is possible when only the crack region can be modeled with PD [32]. Based on current ideas, Madenci [25] extended the BBPD by introducing additional rotation degree of freedom to the PD bonds. In BBPD with bond rotation theory, the PD bond forces depend on the PD bond stretch *s* value and also on the bond rotation vector ***r***:

(5) $$f_{xx^{\prime }}=cs_{xx^{\prime }}\frac{\xi_{xx^{\prime }}}{\left|\xi_{xx^{\prime }}\right|}+dr_{xx^{\prime }}$$

where *d* is the PD bond rotational stiffness. BBPD with bond rotation kinematics is shown in Figure 5.

BBPD bond $\xi_{xx^{\prime }}$ rotation vector ***r_xx’_*** can be expressed from the PD vectors shown in Figure 5 by using an infinitely small rotation tensor $\omega \left(x\right)=\frac{1}{2}\left(\nabla u-u\nabla \right)$:

(6) $$r_{xx^{\prime }}=\frac{\left(\left(u^{\prime }-u\right)-(u^{\prime }-u)\cdot n_{xx^{\prime }}\right)\cdot n_{xx^{\prime }}-\omega (x)\xi_{xx^{\prime }}}{\left|\xi_{xx^{\prime }}\right|}$$

**Figure 6:**
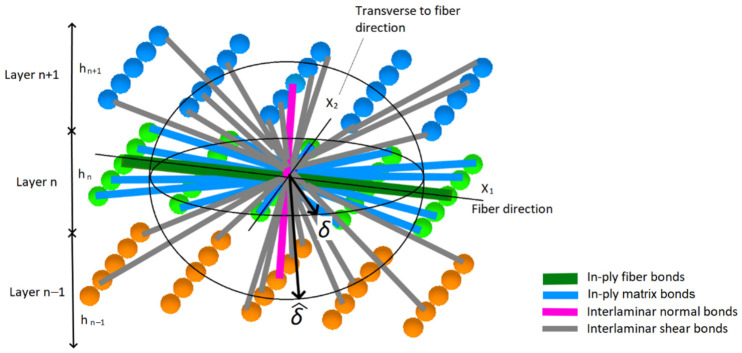
BBPD composite peridynamics.

Due to composite anisotropy, different stiffness PD bonds should be used in the composite PD model [22]: in plane fiber and matrix, normal interlaminar and shear interlaminar, as shown for the BBPD case in Figure 6.

The stiffness of each composite lamina PD bond is derived by comparing classical laminate theory (CLT) and PD strain energy densities according to the PD model formulation, and can be found in [22]. In the case of the current composite laminate BBPD with rotation model, which has not been proposed in the literature yet, the new analytic equations should be derived. Alternatively, the PD bond constant values can be found by comparing the CLT and PD strain energy densities in optimization software, as it is defined in Section 2.4.2. Static failure of the composite BBPD with rotation is based on two criteria: critical stretch *s_c_* value and critical rotation angle $\gamma_{c}$ value (for bonds having a rotational feature and refers to shear failure).

#### 2.3.2. Fatigue Modeling Under Peridynamics

Similar to static failure, fatigue failure can also be introduced to the PD model, calculating the “remaining life” parameter for each PD bond [33]. Nevertheless, this requires a number of cycles-stretch curves of the material. Alternatively, the kinetic theory of fracture (KTF), the atomistic approach, was also successfully introduced into PD models [20,23,34]. The KTF is defined by Equation (7), which computes fatigue damage $n_{xx^{\prime }}$ of PD bond $\xi_{xx^{\prime }}$ relying on maximum stress $\sigma_{max}$ of this PD bond (average stresses of the PD points, connecting that bond), stress ratio *R*, temperature *T*, and loading frequency *f*:

(7) $$n_{xx^{\prime }}\left(N,\sigma_{max},R,T,f\right)=n_{0xx^{\prime }}-{\left\{{\left(n_{0xx^{\prime }}-n_{Ixx^{\prime }}\right)}^{1-\lambda }-(1-\;\lambda )\frac{{\left(kT\right)}^{2}}{h}\frac{N}{\gamma f\sigma_{max\_xx^{\prime }}(1-R)}e^{-\frac{U}{kT}}[e^{\frac{\gamma \sigma_{\max\_xx^{\prime }}}{kT}}-e^{\frac{\gamma R\sigma_{max\_xx^{\prime }}}{kT}}]\right\}}^{\frac{1}{1-\lambda }};\;\mathrm{if}\lambda \neq 1$$

**Figure 7:**
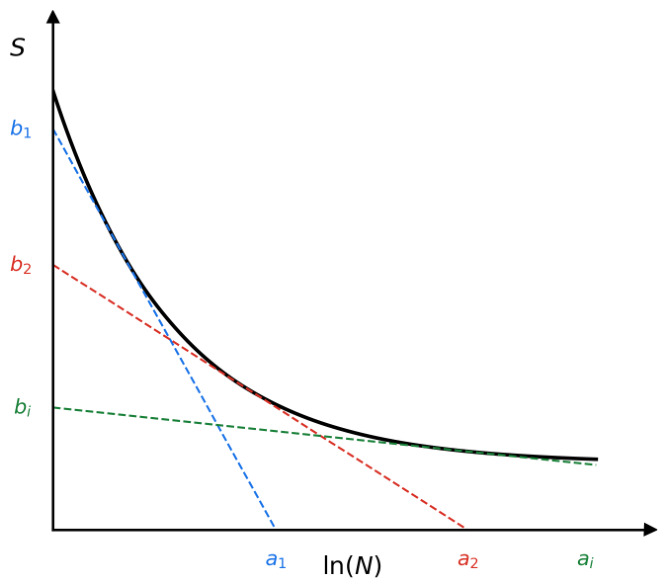
Schematic for determining KTF parameters *U* and γ through the piecewise linear approximation of the S-N curve.

Here, *U*, γ are material parameters, namely activation energy and activation volume, and *λ* is the shape parameter, which defines the rate of damage accumulation over time until final fracture [35]. Activation energy and activation volume can be related to the material S-N curve at specific ranges by following equations [34], as also shown in Figure 7.

(8) $$U=-kT\left(\frac{b_{i}}{a_{i}}+ln\left(f\tau_{0}\right)\right)$$

(9) $$\gamma =-\frac{kT}{a_{i}}$$

Here *a_i_*, *b_i_* are the tangent lines of the S-N curve values at ln(*N*) and *S* axis, *k* = 8.314 kJ/(mol-K) is the Boltzmann constant, $\tau_{0}$ = 10^−13^ s is the characteristic period of the oscillation of atoms. Damage variable $n_{xx^{\prime }}\;=\;0$ indicates no fatigue damage and $n_{xx^{\prime }}\;=1$ complete fatigue damage and failure of the PD bond $\xi_{xx^{\prime }}$. When considering relatively low-level stress cyclic loading of a composite structure, it is assumed that matrix cyclic damage occurs in the structure. Madenci [23] proposed to use effective PD bond stress, computed from normal to fiber stresses $\sigma_{22}$ and shear stresses $\tau_{12}$ as $\sigma_{ef}=\sqrt{{\sigma_{22}}^{2}+{\tau_{12}}^{2}}$ for composite fatigue analysis under the KTF-PD approach. Because strains and stresses are not included in the PD equation of motion (1), additional calculations must be applied. Madenci proposed the PD differential operator (PDDO) [36], which also does not contain conventional derivatives, but is based on integrals and thus valid for the case of discontinuities. The PDDO is used to find the strain gradient from the material point displacement data:

(10) $$F=\left[\int_{H_{x}}\omega \langle \left|\xi \right|\rangle \left(\underline{Y}\left(\xi \right)⨂\xi \right)dV_{\xi }\right]\cdot {\left(\int_{H_{x}}\omega \langle \left|\xi \right|\rangle \left(\xi ⨂\xi \right)dV_{\xi }\right)}^{-1}$$

Then the CCM formulations can be applied to find the strain and stress tensors of material point *x*:

(11) $$E=\frac{1}{2}\left(F^{T}+F+F^{T}\cdot F\right)$$

(12) $$\sigma_{ij}=\sum_{k=1}^{3}\sum_{l=1}^{3}C_{ijkl}e_{kl},i,j=1\ldots 3$$

**Figure 8:**
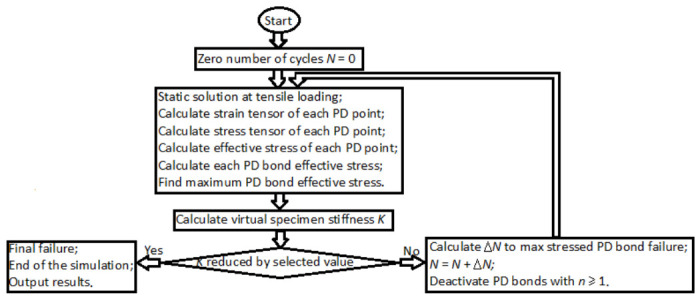
Fatigue damage modeling schematics under the KTF-PD approach.

Effective stresses based on Equations (10)–(12) are also computed in the current study and used to determine the number of cycles to each PD bond failure. KTF-PD simulation is performed by iterations, shown in the schematics given in Figure 8.

Simulation starts with initializing 0 cycles. Then, the first static PD simulation at maximum positive cycle loading is applied. After each static simulation, specimen stiffness *K* is calculated for tracking stiffness reduction and indicating fatigue failure. Later, Equation (7) is applied to find the number of cycles ∆N to the PD bond, experiencing maximum effective stresses. After incrementing the ∆N cycles, fatigue damage variable $n_{xx^{\prime }}$ is computed for each PD bond according to Equation (7). PD bonds with $n_{xx^{\prime }}=1$ are removed. After removing the PD bonds with $n_{xx^{\prime }}=1$, static simulation is run again, and the cycle is repeated. If the virtual specimen stiffness value, computed after static PD simulation, is found to be reduced by the prescribed percent, fatigue failure is considered, and the KTF-PD simulation is finished. The damage $\varphi_{x}$ in the PD model is defined as the ratio of broken PD bonds to total PD bonds in the point’s *x* horizon:

(13) $$\varphi_{x}=\frac{\mathrm{Broken}\;\mathrm{PD}\;\mathrm{bonds}\;\epsilon \;H_{x}}{\mathrm{Total}\;\mathrm{PD}\;\mathrm{bonds}\;\epsilon \;H_{x}}$$

### 2.4. Numerical Modeling of the CFRP Specimen with Notch

#### 2.4.1. FE-AI Applications

To enable quantitative defect identification from full-field surface strain measurements, a machine learning (ML) framework was developed based on synthetic datasets generated using a validated finite element (FE) model.

A three-dimensional FE model of the CFRP coupon was developed in ANSYS APDL 2025 R2. The model dimensions (40 × 20 × 2 mm) were selected to match the region of interest (ROI) used for strain-field analysis. The laminate was represented by six individual solid ply volumes with stacking sequence [(0/90)°/90°/0°]s, where both woven and unidirectional plies were assigned orthotropic elastic properties and appropriate local material coordinate systems.

**Figure 9:**
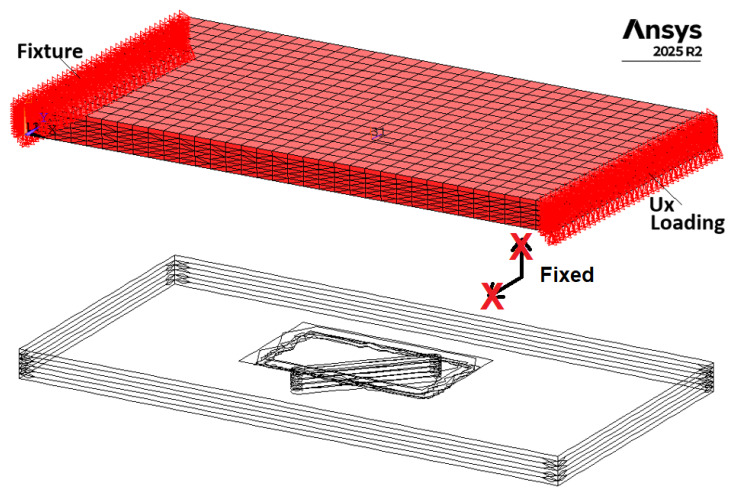
FEM of the CFRP specimen with different notch dimensions for AI training.

In the experimental setup, two notches were introduced in a single specimen to enable simultaneous tracking of damage evolution. However, for numerical efficiency and model simplicity, only a single notch was considered in the FE model (Figure 9). This assumption is supported by additional comparisons between two-notch and corresponding single-notch FE models, which show that the strain localization in the vicinity of each notch is dominated by local geometry, with negligible interaction effects between the notches at the considered spacing. This simplification does not affect the local stress–strain state in the vicinity of the notch and therefore does not influence the AI training data. The FE model validation was performed by comparing the simulated and experimentally measured (3D DIC) strain fields at the initial state (*N* = 0), confirming good agreement in terms of strain localization and distribution patterns in the vicinity of the notch. The notch was modeled as a rotated obround slot with its geometry defined by three parameters: notch depth (NOT_D), notch orientation angle (NOT_ANG), and fixed notch length and width.

Internal delamination was introduced beneath the notch by defining local interlaminar contact interfaces between plies 4 and 5. The location of the delamination interface was selected based on ultrasound inspection results. Within the delaminated region, surface-to-surface contact was defined, while the surrounding intact interface was modeled using bonded contact. This approach allowed controlled parametrization of the defect footprint. The delamination was centered beneath the notch and characterized primarily by its in-plane length (DEL_L), with the width defined as DEL_W=DEL_L/2.

The specimen was subjected to tensile loading by prescribing a uniform axial displacement corresponding to the experimental load amplitude of F=6 kN. The opposite end was constrained using an auxiliary constraint to prevent rigid body motion while allowing free transverse contraction (Figure 9). A nonlinear static analysis with geometric nonlinearity enabled was performed. For each simulation, elastic strain fields were extracted from the top surface ROI corresponding to the DIC measurement area. To minimize boundary condition effects, the ROI (38 × 18 mm) was selected slightly smaller than the FE model domain.

To ensure consistency between simulations and experiments, the FE model was validated against 3D DIC strain fields measured at selected cycle counts under peak cyclic loading. Following validation, a parametric study was performed using a PyMAPDL-0.72.0 based workflow, in which notch depth, notch angle, and delamination size were varied according to a predefined design-of-experiment (DOE) plan, resulting in a dataset of 109 simulations. It should be noted that the dataset size is relatively limited; therefore, the developed models are intended to demonstrate the feasibility of the proposed approach and comparative performance of different ML methods, rather than providing fully generalized predictive capability.

The DOE plan included three parameters: notch depth (0.8–1.67 mm, discrete levels), notch angle (0–90°), and delamination length (0–30 mm, with width defined as half of the length). A hybrid sampling strategy was adopted, combining uniform sampling of notch depth with stratified random, conceptually similar to Latin hypercube sampling of notch angle and delamination length to ensure uniform coverage of the parameter space. The sampled angle and delamination values were then randomly paired within each depth level, ensuring coverage of the parameter space without introducing artificial correlations.

For each case, the three surface strain components ($\varepsilon_{x}$, $\varepsilon_{y}$, $\gamma_{xy}$) were extracted and interpolated onto a regular grid consistent with the DIC data structure.

To convert high-dimensional strain fields into a compact and informative representation, a two-level feature extraction strategy was applied. First, global statistical descriptors of the strain components were computed over the entire ROI. Second, to capture the spatial localization of deformation patterns, the strain field was divided into a regular grid of 4×8 segments (32 subregions). Within each segment, localized features were extracted, including mean, minimum, and maximum strain values, as well as strain-energy-like quantities defined as $\varepsilon_{x}^{2}+\varepsilon_{y}^{2}+\gamma_{xy}^{2}$ and corresponding strain magnitude statistics.

Each simulated case was labeled using the known FE input parameters. The resulting dataset was used to train ML models for multi-stage inference, including: (i) defect presence detection, (ii) defect type classification, and (iii) quantitative estimation of defect parameters. Specifically, the following prediction tasks were defined: binary classification (defect presence), multi-class classification (defect type: none, notch, delamination, combined), and regression tasks (notch depth, notch angle, and delamination size expressed as length or area).

A set of supervised ML algorithms was evaluated, including Logistic Regression, Support Vector Machines with radial basis function kernel, Random Forest, Gradient Boosting, Ridge regression, Support Vector Regression, and Gradient Boosting Regressor. All models were implemented using standardized pipelines incorporating median-based missing value imputation, optional feature scaling, and model-specific training.

To assess the importance of spatial information, four feature configurations were investigated: (i) global strain descriptors, (ii) segment-based basic statistics, (iii) segment-based energy-related features, and (iv) a combined segment-based feature set. Model performance was evaluated using cross-validation, with stratified 5-fold cross-validation applied for classification tasks and standard 5-fold cross-validation for regression tasks. Performance metrics included accuracy, balanced accuracy, and macro F1-score for classification, and mean absolute error (MAE), root mean square error (RMSE), and coefficient of determination ($R^{2}$) for regression. Balanced accuracy and MAE were selected as primary evaluation metrics due to their robustness and physical interpretability.

#### 2.4.2. PD Model

**Figure 10:**
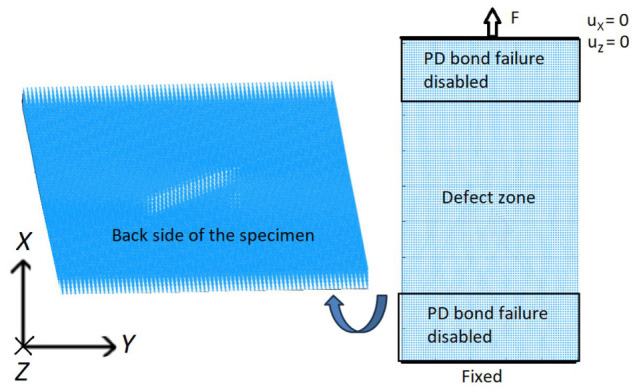
BBPD with bond rotation PD model of the CFRP specimen with notch.

PD model of the CFRP specimen with a notch was created using discretized BBPD with bond rotation theory. The same as for FEM, only one notch was created. In order to reduce computational time, only a 45 mm length specimen segment was modeled. To capture a notch in the specimen geometry, the PD grid size was selected *Δx* = 0.33 mm, resulting in 3 points per notch width and 75 points per specimen width. A deeper notch (depth of 1.5 mm) was included in the specimen PD model. Standard PD horizon size of *δ* = 3.1 *Δx* was selected. The notch was modeled as missing PD points and PD bonds in the notch zone. In order to prevent possible cyclic damage at the boundaries in the PD model, PD bond failure was deactivated for the PD bonds at the boundaries, as shown in the model schematics in Figure 10.

**Figure 11:**
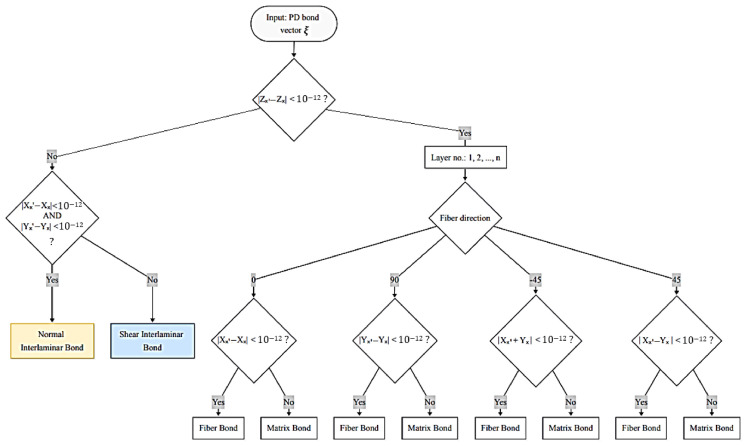
PD bonds classification flowchart according to the PD bond direction.

Boundary conditions in the model (Figure 10) are the same as during the experiment: a completely fixed bottom of the virtual specimen and top of the specimen are allowed to move in the *X* direction while loaded by an external force *F* in the *X* direction. Relative position vector $\xi_{xx^{\prime }}$ between PD points x and x’ is given by $\xi_{xx^{\prime }}=[X_{x^{\prime }}-X_{x};Y_{x^{\prime }}-Y_{x};Z_{x^{\prime }}-Z_{x}]$, whose components are employed to identify the PD bond direction and then the PD bond type: fiber, matrix, in-plane shear, or interlaminar PD bond. The classification algorithm for PD bonds, based on the PD bond orientation and the fiber direction within each composite layer, is illustrated in Figure 11. This algorithm is tailored for composite layups of 0°, ±45°, and 90°, which are used in composites in this study. A numerical tolerance of 10^−12^ is implemented when calculating coordinate differences; this threshold is sufficient to ensure the robust and accurate assignment of fiber bonds while accounting for potential floating-point precision errors.

**Figure 12:**
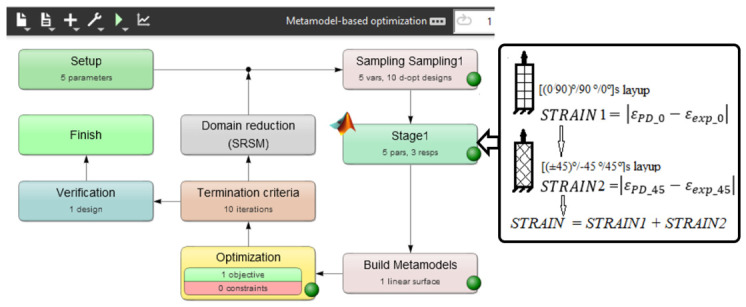
BBPD with bond rotation model of the CFRP specimen material parameters calibration setup.

In order to establish proper different PD bond stiffness, a model linear elastic behavior was first calibrated from experimental tensile tests of the CFRP specimens made of the CFRP plate. [(0/90)°/90°/0°]s and [(±45)°/−45°/45°]s layups configurations were considered to calibrate the PD model to ensure agreement for both layups’ experimental results. Because the specimen model must be computed multiple times to find the proper PD bond parameters, PD model fragments of 15 × 45 × 6 points, having identical boundary conditions to Figure 12, were used in order to reduce the calibration time.

Optimization software LS-Opt 2023 R1 was used to run two in series PD simulations of a specimen with layups of [(0/90)°/90°/0°]s and [(±45)°/−45°/45°]s. The effective stiffness of each layup specimen, loaded by a constant force, was evaluated by computing tensile strains $\varepsilon_{PD\_0}$ and $\varepsilon_{PD\_45}$. The same strains $\varepsilon_{exp\_0},\varepsilon_{exp\_45}$ at the same loading were taken from experimental tensile curves. Finally, the parameter

(14) $$STRAIN=\left|\varepsilon_{PD\_0}-\varepsilon_{exp\_0}\right|+\left|\varepsilon_{PD\_45}-\varepsilon_{exp\_45}\right|$$

was computed. By selecting different combinations of the PD bond stiffness in LS-Opt, the optimization objective was to minimize the parameter *STRAI*, ensuring the best possible agreement between the PD and experimental elastic behavior of [(0/90)°/90°/0°]s and [(±45)°/−45°/45°]s layups specimens. A maximum of 10 iteration criteria was set. Automatic variables range reduction for each iteration (“Domain reduction” in Figure 12) was included. The general calibration setup in the LS-Opt window is shown in Figure 12.

## 3. Results and Discussion

### 3.1. Cyclic Tests of CFRP Specimen with Notch

**Figure 13:**
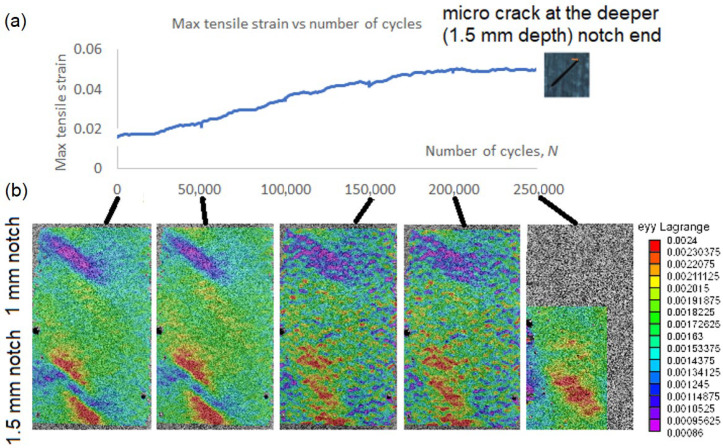
CFRP specimen with notches cyclic test results: (**a**) Stiffness reduction with the number of cycles; (**b**) DIC measured surface strain at different numbers of cycles.

A cyclic test of the CFRP specimen with notches was run up to 250,000 cycles. No heating, which could affect the specimen material properties, was observed during the test. The damage growth was not visible on the specimen during the test, although stiffness reduction in the specimen, seen in the number of cycles–displacement plot (Figure 13a), was detected. The stiffness of the specimen dropped about 25% after 250,000 cycles. The DIC surface strain in tensile direction images (also given in Figure 13b) were taken at the initial moment and after every 50,000 cycles.

It should be noted that quantitative information about the defect state was available only at two stages of the experiment: at the initial state (*N* = 0), where the specimen contained two predefined notches (10 × 1.0 × 1.0 mm and 10 × 1.0 × 1.5 mm), and after the fatigue test was stopped at 250,000 cycles, when a post-test inspection revealed a micro-crack of approximately 1 mm length at the deeper notch. No direct quantitative measurements of damage evolution were available during intermediate loading stages.

Minor changes in the strain images were detected after 50,000 cycles and significant changes after 150,000 cycles, as shown in Figure 13b. With an increasing number of cycles, the region of elevated strain values expanded, indicating progressive damage accumulation.

To support this observation, quantitative analysis of the strain fields was performed by evaluating statistical descriptors within the region of interest. While the peak strain values remained nearly constant (variation within approximately 5%), a consistent increase in the spatial extent of high-strain regions was observed. This indicates redistribution of strain rather than local amplification, which is characteristic of stiffness degradation.

The evolution of strain fields also showed a clear dependence on notch depth. The strain field around the deeper notch (1.5 mm) exhibited a more pronounced increase in the area of elevated strain and stronger gradients compared to the shallower notch (1.0 mm). In contrast, no significant changes in strain distribution were observed around the 1.0 mm notch, indicating the absence of detectable damage growth in that region.

The observed increase in the spatial extent of high-strain regions correlates with the measured global stiffness reduction and the final detection of a micro-crack at the deeper notch. These results confirm that DIC strain fields provide quantitative indicators of damage evolution, particularly in terms of damage localization and progression. However, the results also show that the peak strain magnitude alone is not sufficient to characterize the damage state, and parameters such as strain distribution and affected area must be considered.

**Figure 14:**
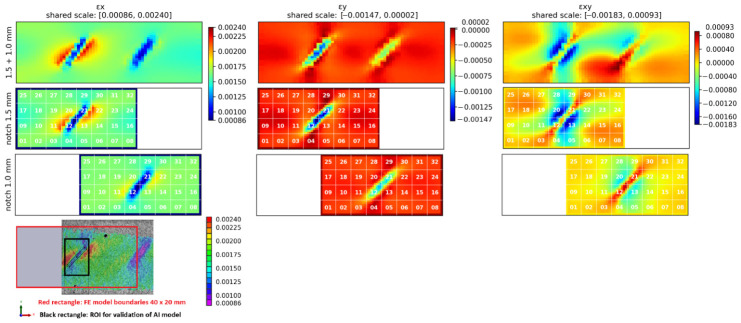
Comparison of strain fields used for model validation: (top row) FE model with two notches; (second and third rows) FE models with single notches of 1.5 mm and 1.0 mm depth; (bottom row) experimentally measured DIC longitudinal strain field. A consistent color scale is used for comparison. The red rectangle indicates the FE model domain, the black rectangle indicates the ROI used for analysis.

To further support the correlation between strain fields and damage evolution, a comparison between FE simulations and experimental DIC measurements was performed (Figure 14). The first row shows the FE strain fields for a specimen with two notches, corresponding to the experimental configuration. The second and third rows present FE results for simplified single-notch models with depths of 1.5 mm and 1.0 mm, respectively, while the fourth row shows the experimentally measured DIC longitudinal strain field at the initial state. A consistent strain scale was used for all images to allow direct comparison.

The comparison in Figure 14 shows good agreement between FE and DIC results in terms of strain localization, orientation, and relative distribution of strain concentrations. The deeper notch (1.5 mm) is associated with a larger area of elevated strain and steeper strain gradients compared to the shallower notch (1.0 mm), which is consistently observed in both FE and experimental data. At the same time, the peak strain values remain within a similar range, indicating that damage evolution is primarily reflected by changes in the spatial distribution of strain rather than by an increase in maximum strain.

These results confirm that the simplified single-notch FE model adequately reproduces the strain field characteristics observed experimentally and can be used for subsequent parametric studies and ML dataset generation.

### 3.2. Ultrasound Inspection Results

**Figure 15:**
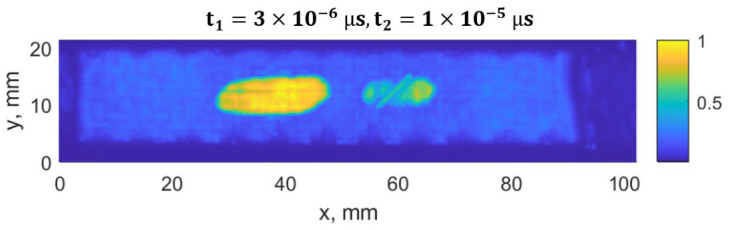
C-scan (top view) of the CFRP sample. On the left side, the notch depth is 1.5 mm, on the right side, 1.0 mm.

**Figure 16:**
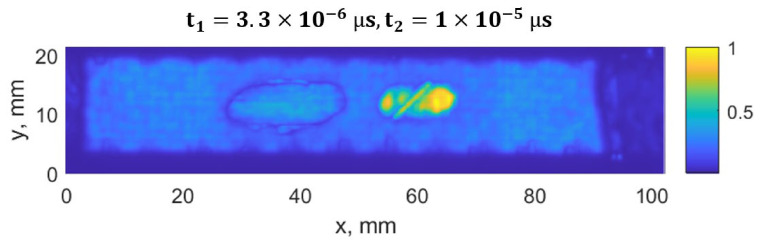
C-scan (top view) of the CFRP sample using a time window of t_1_ = 3.3 µs to t_2_ = 10 µs, showing the defect located closer to the backwall. On the left side, the notch depth is 1.5 mm, on the right side, 1.0 mm.

**Figure 17:**
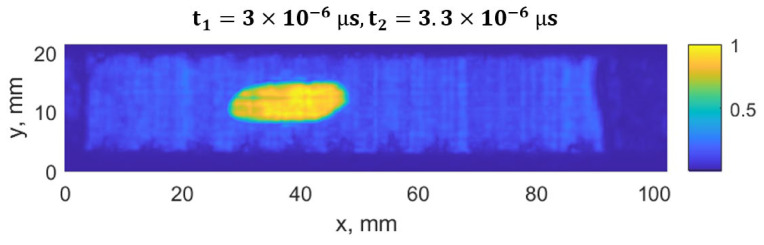
C-scan (top view) of the CFRP sample using a time window of t_1_ = 3.0 µs to t_2_ = 3.3 µs, showing the defect located close to the surface. Only on the left side, visible delamination corresponds to the initial defect, a notch with a depth of 1.5 mm.

**Figure 18:**
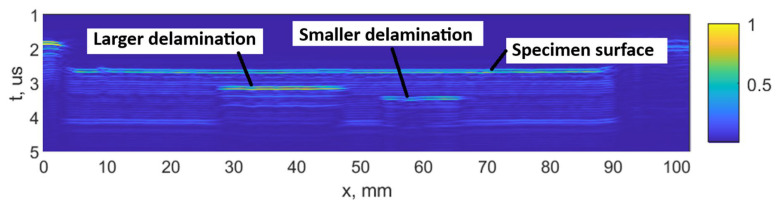
B-scan of the CFRP sample at *y* = 11 mm, indicating the depth of the defects. On the left side, the notch depth is 1.5 mm, on the right side, 1.0 mm.

The damage growth around both notches was visible as delamination detected by ultrasound scans, presented in Figure 15, Figure 16, Figure 17 and Figure 18. It was estimated that a large delamination area around the notch of depth of 1.5 mm is 115.1 mm^2^, depth below the surface is approx. 0.65 mm; whereas small delamination around the notch of depth of 1 mm area is 56.49 mm^2^, and depth below the surface is approx. 1.1 mm.

In Figure 15, a C-scan image of the sample is shown, showing all the defects in the specimen at different depths. In Figure 16 and Figure 17, C-scan images at different depths of the specimen are shown, visualizing the delamination that occurred at different depths. In Figure 18, a B-scan image of the specimen is presented, showing the depths of the delamination detected according to the surface of the specimen. A smaller delamination formed around the notch of 1 mm depth, visible in all the images on the left. Larger delamination formed around the notch of 1.5 mm depth.

The ultrasound measurements were used to quantify the delamination area and depth after fatigue loading and to confirm the relationship between strain field changes and damage/delamination size. These values were then used to define representative defect configurations in the FE simulations for ML dataset generation.

### 3.3. AI-Based Defect Identification and Quantification

The developed machine learning (ML) framework was applied to a dataset consisting of 109 FE-generated samples, covering a wide range of defect configurations defined by notch depth, notch orientation angle, and delamination size. The ML learning model data are summarized in Table 2 where the best performing models are highlighted in bold.

The results demonstrate that full-field surface strain data contain sufficient information for reliable defect identification and quantitative characterization. The best-performing models achieved perfect notch detection (balanced accuracy = 1.00), high delamination detection accuracy (up to 0.85), and low regression errors, reaching 0.039 mm for notch depth, 3.17° for notch angle, and 3.02 mm for delamination length.

**Defect Detection and Classification**. Binary classification results indicate that defect presence can be robustly identified using strain-based descriptors alone. Notch detection achieved near-perfect performance across all feature sets, with Random Forest models reaching a balanced accuracy of 1.00 when segment-based features were used. Even global descriptors provided high accuracy (0.995), indicating that notch-induced strain concentrations are strongly pronounced and globally detectable.

In contrast, delamination detection proved more challenging, with balanced accuracy values ranging from 0.77 to 0.86. The lower performance reflects the more distributed and less localized strain signatures associated with interlaminar defects. Interestingly, global strain descriptors slightly outperformed segment-based features for this task, suggesting that delamination effects are better captured through global deformation patterns rather than strictly localized indicators.

**Effect of Feature Representation**. Feature representation plays a critical role in model performance. While global strain statistics provide a strong baseline, segment-based features consistently improved predictive accuracy for most tasks, particularly for regression problems.

The most significant improvements were observed for notch parameter estimation. For example, the prediction error for notch depth decreased from 0.062 mm (global features) to 0.039 mm when using fully segmented features, with a corresponding increase in $R^{2}$ from 0.923 to 0.976.

Energy-based features alone were generally less effective compared to combined or basic segment descriptors. This suggests that while strain energy captures important deformation characteristics, it does not fully represent the directional and localized strain variations required for accurate defect characterization.

The results confirm that spatial segmentation enables the extraction of physically meaningful features that reflect localized strain gradients, which are critical for distinguishing between defect types and quantifying defect parameters.

**Regression of Defect Parameters**. The regression results reveal different levels of sensitivity of strain fields to defect parameters. Notch depth was predicted with the highest accuracy, reflecting its direct influence on localized strain magnitude. The strong correlation between strain concentration and defect depth enables highly accurate inverse identification.

Notch orientation angle was predicted with moderate accuracy, with errors around 3–5°, indicating a more complex and nonlinear relationship between orientation and strain distribution. The best results were obtained using segment-based features combined with both linear (ridge) and nonlinear (gradient boosting) models, suggesting that different aspects of the strain field contribute to orientation sensitivity.

Delamination size estimation showed moderate accuracy, with errors around 3 mm and $R^{2}$ values below 0.80. This is consistent with the diffuse nature of delamination-induced strain fields, which reduces sensitivity and increases uncertainty in inverse identification.

**Figure 19:**
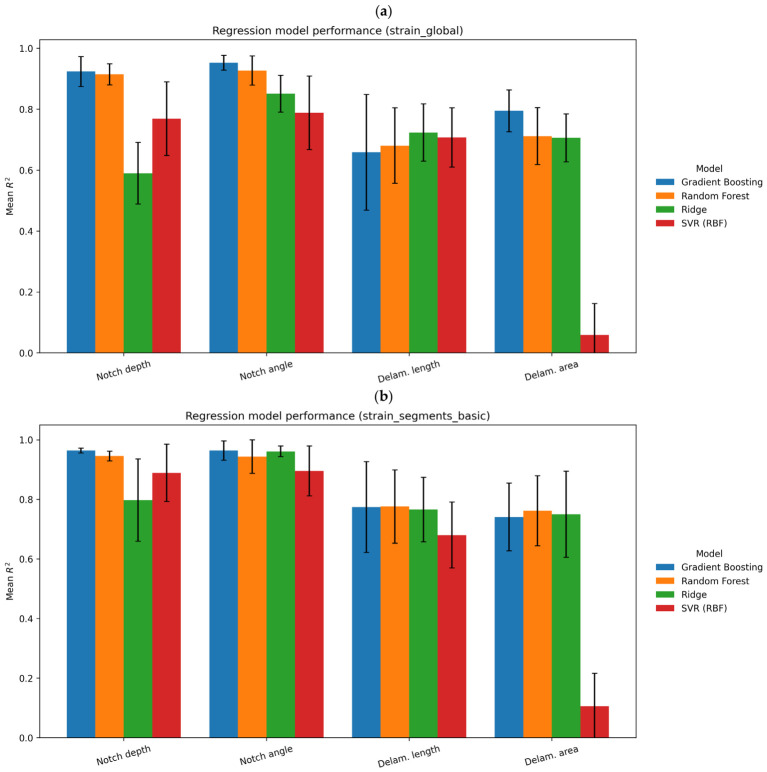
Regression model performance under the global strain (**a**) and strain segments (**b**).

Across all regression tasks, ensemble-based models (Gradient Boosting and Random Forest) consistently outperformed linear models, confirming the nonlinear nature of the mapping between strain-derived features and defect parameters (Figure 19).

The spatial segmentation of the strain field into 32 regions used for feature extraction was illustrated in Figure 14. The dominance of segment-localized features confirms that defect identification is governed primarily by spatial strain gradients rather than global strain levels.

For notch defects, the most influential features are concentrated in segments located near the defect region, corresponding to localized strain peaks. In contrast, delamination-related features are more broadly distributed, reflecting the smoother strain gradients associated with interlaminar damage.

This observation provides strong physical validation of the proposed approach, demonstrating that the ML models capture meaningful deformation mechanisms rather than purely statistical correlations.

As reflected in Table 2, the reduced performance for delamination-related tasks (balanced accuracy up to 0.857 and R^2^ below 0.80) is attributed to the diffuse nature of interlaminar damage. Unlike notch defects, which produce localized strain concentrations, delamination induces smoother and more spatially distributed strain variations, reducing the sensitivity of surface strain features to defect size. This results in increased uncertainty in inverse identification and highlights a fundamental limitation of strain-based SHM for interlaminar damage quantification.

**Validation Using Experimental DIC Data**. To assess the predictive capability of the trained models, experimental DIC strain fields from Figure 14 were used as input for validation. To exclude potential bias, FE cases corresponding exactly to the experimental specimen parameters were removed from the training dataset, and the models were retrained, yielding comparable performance and confirming the robustness of the results.

The three strain components $\left(\varepsilon_{x},\varepsilon_{y},\gamma_{xy}\right)$ measured at the initial stage of cyclic loading were interpolated onto a regular 64×64 grid, consistent with the synthetic dataset structure. The best-performing models identified in Table 2 were subsequently applied. The prediction results are summarized in Table 3.

The validation results confirmed the robustness of the approach. Both classification models correctly identified the presence of notch and delamination defects (prediction = 1.0), demonstrating successful transfer from synthetic FE-generated data to experimentally measured DIC strain fields.

Quantitative predictions are in good agreement with the expected defect characteristics. The predicted notch depth (1.32 mm) and orientation angle (36.7°) are close to the actual values of the tested specimen (1.5 mm and 45°, respectively). Similarly, the predicted delamination length (2.24 mm) and area (12.6 mm^2^) indicate the presence of a relatively small interlaminar defect, consistent with early-stage damage development.

Despite the limited size of the validation dataset, these results demonstrate that the ML framework trained exclusively on synthetic FE data is capable of generalizing to experimental measurements. This confirms the feasibility of combining physics-based simulations with full-field DIC data for solving inverse defect identification problems in composite structures.

Overall, the results demonstrate that full-field strain measurements, combined with physically informed feature extraction and classical ML models, provide a reliable and interpretable solution for defect identification and quantification.

Spatial segmentation of strain fields plays a key role in capturing localized deformation patterns and significantly enhances model performance. Importantly, the proposed approach avoids the need for computationally intensive image-based deep learning methods, while maintaining high accuracy and interpretability.

The successful validation using experimental DIC data further highlights the potential of the approach for real-world structural health monitoring applications. The methodology enables non-contact, data-driven defect detection and characterization, forming a foundation for future integration with predictive models for damage evolution and remaining life assessment.

### 3.4. Numerical Modeling with Peridynamics

PD model elastic properties calibration in LS-Opt took 6 h of computational time. The optimization objective of 0.0037 was achieved after 10 iterations. The calibrated CFRP composite PD bond stiffness for the current model is given in Table 4.

**Figure 20:**
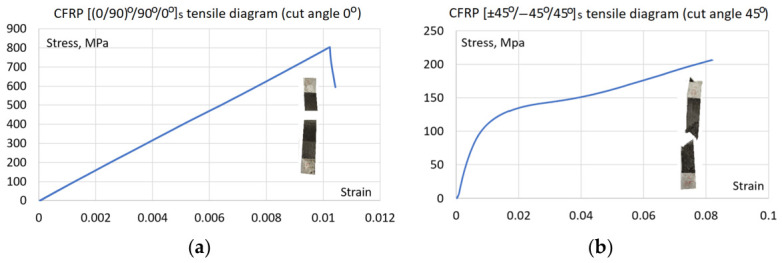
Experimental tensile curves of the [(0/90)°/90°/0°]s (**a**) and [(±45)°/−45°/45°]s (**b**) layup specimens.

**Figure 21:**
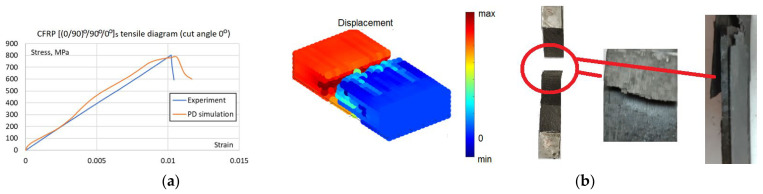
BBPD with bond rotation PD model static properties calibration: (**a**) [(0/90)°/90°/0°]s layup specimen tensile curves; (**b**) Failure comparison, the best visible in simulated displacement plot.

**Figure 22:**
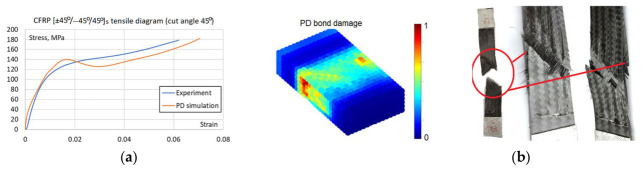
BBPD with bond rotation PD model static properties calibration: (**a**) [(±45)°/−45°/45°]s layup specimen tensile curves; (**b**) Failure comparison, the best visible in simulated PD damage plot.

Although cyclic damage is the focus, static failure remains integrated into the model to account for the possible final static failure after the cyclic damage accumulation. Failure parameters were calibrated manually, as this allowed for a simultaneous assessment of both numerical accuracy and failure morphology, a qualitative validation that automated optimization cannot capture. Initial estimates of fiber and matrix critical stretch *s_fc_* = *s_mc_* = 0.095 and critical matrix bonds rotation angle *γ_mc_* = 0.011 were derived from a non-failure model at the failure stress levels taken from the experimental tensile curve. Then the parameter values were iteratively refined within a range of ±15%. The simulation of [(0/90)°/90°/0°]s layup specimen served to determine critical stretch values, while [(±45)°/−45°/45°]s layup served to determine critical rotation angle and check the matrix critical stretch value. After five iterations, the final parameters of critical stretch *s_fc_* = *s_mc_* = 0.0085 and critical rotation angle of *γ_mc_* = 0.012 were established, ensuring that simulated failure modes and tensile curves (Figure 20, Figure 21 and Figure 22) match experimental observations. The resulting error between the simulated and experimental failure strains at equivalent stress levels was less than 5% for the [(0/90)°/90°/0°]s specimen, which is sufficient because the primary objective of the model is to capture cyclic damage evolution rather than purely static failure.

Despite thorough parameter refinement, the maximum discrepancy in failure strain values for [(±45)°/−45°/45°]s layup specimen (Figure 22) is 13%. It should be noted that due to the fixed in-plane shear modulus inherent in the BBPD with the bond rotation model [25], achieving simultaneous perfect agreement for all layups is constrained. Nevertheless, the BBPD model with bond rotation offers superior accuracy compared to the conventional BBPD, which is limited by a fixed Poisson’s ratio. Furthermore, a key advantage of this approach is its compatibility with existing FE, unlike SBPD, which, despite its high accuracy, remains challenging to implement within existing FE frameworks. Notably, despite the quantitative differences between the curves, the BBPD model successfully replicated both the fiber failure mode in the specimen (Figure 21b) and the shear failure mode in the specimen (Figure 22b).

**Figure 23:**
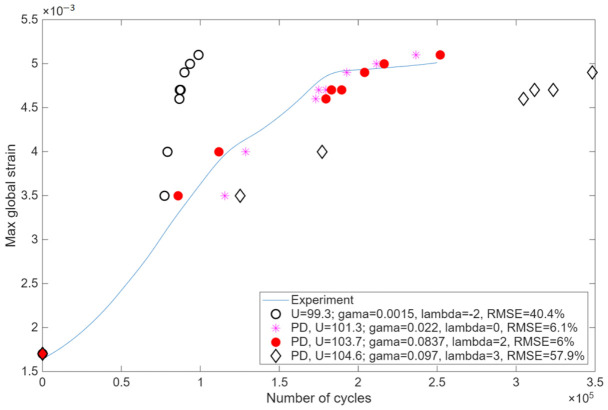
Cyclic PD model calibration: experimental and PD simulated max strain vs number of cycles data.

Because the current cyclic loading maximum load is 15% specimen failure load (6000 N vs. 40,000 N), the cyclic damage mechanism is considered as matrix, but not fiber failure in the specimen. Madenci proposed [23] that effective stresses $\sigma_{ef}=\sqrt{{\sigma_{22}}^{2}+{\tau_{12}}^{2}}$ were computed and used to predict the fatigue life of each PD bond. Due to the reason that specific S-N curves for the OKE CFRP specimens were unavailable and that generic literature data often fail to account for layup-specific damage evolution, the KTF parameters were identified using an inverse calibration method. Cyclic PD simulation, consisting of a set of static PD simulations to compute effective stresses after each PD bond cyclic failure, was initially run by trial fatigue parameters, namely activation energy *U*, activation volume *γ* and damage accumulation parameter λ. After the simulation was finished, the number of cycles was computed. Initially, the specimen stiffness reduction was in good agreement with the experimental result (Figure 23), although the number of cycles was different by several times from the experiment. Then, the PD model cyclic calibration was performed to find the best fit between experimental and simulated fatigue data of Figure 23. Experimental and PD simulation max strain–number of cycles results were compared by computing the minimum square sum of differences between the experimental and simulated number of cycles. The procedure was performed in MATLAB R2025b with the function *fminsearch* with initial values of *U* = 140 kJ/k-mol, *γ* = 0.012 for the CFRP composite taken from Madenci’s study [23]. The shape parameter λ was taken at discrete integer values in the range from −2 to 9 based on studies [23,35] and for each *λ*, the *U* and the *γ* were optimized. The best fit optimization results and errors are present in Figure 23.

Activation energy of *U* = 103.7 kJ/k-mol, activation volume of *γ* = 0.0837 and damage accumulation parameter λ = 2 resulted in the best agreement between the experimental and PD data, yielding an RMSE of 6% (Figure 23). While different material activation energy *U* and activation volume *γ* values affect the S-N curve of the material, as it was explained through Equations (8) and (9), the effect of different shape parameter λ values is specifically illustrated in Figure 23, showing different damage accumulation rates when λ = −2; 0; 2; 3.

**Figure 24:**
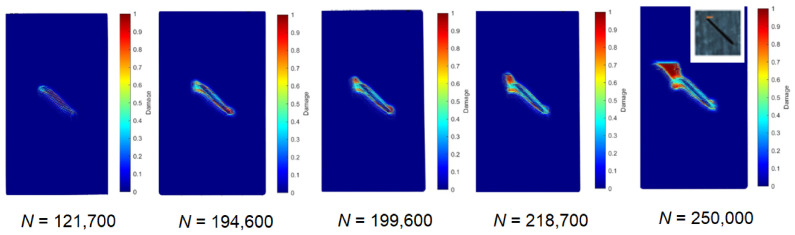
Simulated by the KTF-PD approach, notch growth with increasing number of cycles.

Based on Figure 20, Figure 21 and Figure 22 results, the PD model was completely validated both for material behavior at static loading as well as cyclic loading. The simulated cyclic damage at different numbers of cycles is shown in Figure 24. The plotted damages are the plots of the same damage parameter $\varphi_{x}$ computed according to Equation (13) among different specimen points at different numbers of cycles.

Damage growth around the notch is visible in Figure 24 with the increasing number of cycles. PD model stiffness reduction in the specimen correlates well with experimental stiffness reduction, as visible in Figure 23. Finally, the detected micro-crack near the 1.5 mm depth notch in the real specimen agrees with the simulated damage in Figure 24 after 250,000 cycles. This validates the PD model for the structure fatigue damage growth modeling and fatigue life prediction. Future developments of the current BBPD model will focus on both predictive capabilities and computational efficiency. By extending the model’s calibration to interlaminar behavior, such as static and cyclic delamination growth, it will be possible to simulate the evolution of induced delamination and directly correlate the numerical results with experimental ultrasound scan data. Furthermore, to address the substantial computational demand that requires two weeks for the current simulations, future work will incorporate optimization strategies, such as the cycle jump, multi-GPU [37], and FEM-PD coupling [38], to significantly reduce processing time without compromising accuracy.

## 4. Conclusions

This study presents a physics-informed framework for defect identification, quantification, and damage evolution in CFRP structures by integrating full-field DIC measurements, validated finite element (FE) modeling, machine learning (ML), and peridynamics (PD).

The ML models, trained on FE-generated data, achieved high accuracy for localized defects: notch detection reached a balanced accuracy of 1.00, while notch depth and orientation were predicted with errors of ~0.04 mm (R^2^ = 0.976) and ~3° (R^2^ = 0.968), respectively. In contrast, delamination detection and size estimation were less accurate (balanced accuracy up to 0.857, R^2^ < 0.80), reflecting the diffuse strain signatures of interlaminar damage. Spatial segmentation of strain fields significantly improved predictive performance, confirming the importance of localized strain gradients.

Validation using experimental DIC data demonstrated successful transferability from synthetic to real measurements, with correct identification of defect types and realistic prediction of defect parameters. The PD model reproduced stiffness degradation (~25%) and damage evolution under cyclic loading, capturing micro-crack initiation after ~250,000 cycles.

The calibrated PD model reproduced both static and cyclic material behavior and accurately captured fatigue damage growth near the notch, consistent with experimental observations. The cyclic PD model calibrated using experimentally derived strain–cycle relationships demonstrated good agreement with stiffness degradation and damage evolution observed experimentally. Simulated damage growth around the notch correlated with experimentally observed micro-crack initiation after approximately 250,000 cycles, confirming the capability of the PD model to capture fatigue-driven damage mechanisms. This enables not only damage detection but also the prediction of damage progression and structural lifetime.

Overall, the results show that surface strain-based ML is highly effective for detecting and quantifying localized defects, while delamination can be reliably detected but only coarsely quantified. The proposed ML–PD approach enables non-contact, interpretable structural health monitoring, forming a foundation for physics-informed digital twins and remaining life assessment of composite structures.

## Figures and Tables

**Table 1:** DIC parameters setup used for experimental testing.

Parameter	Value
Step size	11 px (0.09 mm for the current setup)
Subset size	57 px (0.46 mm for the current setup)
Strain filter size	11%

**Table 2:** Best-performing machine learning models for defect identification and quantification.

Task	Task Type	Target	Feature	Number of Features	Model	Primary Metric	Mean RMSE	Mean R^2^
notch_binary	classification	is_notch	strain_global	31	gb_cls	0.995	-	-
notch_binary	classification	is_notch	strain_segments_all	416	**rf_cls**	**1.000**	-	-
notch_binary	classification	is_notch	strain_segments_basic	288	**rf_cls**	**1.000**	-	-
notch_binary	classification	is_notch	strain_segments_energy	128	logreg	0.960	-	-
delamination_binary	classification	is_delamination	strain_global	31	**logreg**	**0.857**	-	-
delamination_binary	classification	is_delamination	strain_segments_all	416	logreg	0.830	-	-
delamination_binary	classification	is_delamination	strain_segments_basic	288	logreg	0.818	-	-
delamination_binary	classification	is_delamination	strain_segments_energy	128	logreg	0.767	-	-
depth_reg	regression	not_d_mm	strain_global	31	gb_reg	0.062	0.105	0.923
depth_reg	regression	not_d_mm	strain_segments_all	416	**gb_reg**	**0.039**	0.062	0.976
depth_reg	regression	not_d_mm	strain_segments_basic	288	gb_reg	0.045	0.075	0.964
depth_reg	regression	not_d_mm	strain_segments_energy	128	gb_reg	0.066	0.124	0.904
angle_reg	regression	not_ang_deg	strain_global	31	gb_reg	4.208	5.783	0.952
angle_reg	regression	not_ang_deg	strain_segments_all	416	**ridge**	**3.173**	4.776	0.968
angle_reg	regression	not_ang_deg	strain_segments_basic	288	gb_reg	3.310	4.833	0.964
angle_reg	regression	not_ang_deg	strain_segments_energy	128	ridge	5.472	8.184	0.908
delam_reg	regression	del_l_mm	strain_global	31	svr_rbf	3.809	5.338	0.707
delam_reg	regression	del_l_mm	strain_segments_all	416	gb_reg	3.080	4.473	0.779
delam_reg	regression	del_l_mm	strain_segments_basic	288	**gb_reg**	**3.023**	4.504	0.774
delam_reg	regression	del_l_mm	strain_segments_energy	128	gb_reg	4.060	5.344	0.700

Notes: RF—Random Forest; GB—Gradient Boosting; Log_reg—Logistic Regression; Primary Metric for cls task is BAcc—Balanced Accuracy; for reg task MAE—Mean Absolute Error. Segment-based features correspond to the spatial partitioning of strain fields into 32 subregions. Bold is indicating best-performing models.

**Table 3:** Predictions of defect presence and parameters obtained from experimental DIC data using the best-performing machine learning models.

Task	Task Type	Target	Feature	Model	Prediction
notch_binary	classification	is_notch	strain_segments_basic	rf_cls	1.000
delamination_binary	classification	is_delamination	strain_global	logreg	1.000
depth_reg	regression	not_d_mm	strain_segments_all	gb_reg	0.039
angle_reg	regression	not_ang_deg	strain_segments_all	ridge	36.74
delam_reg	regression	del_l_mm	strain_segments_basic	gb_reg	2.239

**Table 4:** Material parameters used for PD modeling.

Parameter	Value
Fiber bond stiffness C_MAT1, N/m^6^	1.91 × 10^23^
Matrix bond stiffness C_MAT2, N/m^6^	8.25 × 10^22^
Normal interlaminar bond stiffness, C_MAT3, N/m^6^	3.93 × 10^24^
Shear interlaminar bond stiffness, C_MAT4, N/m^6^	4.89 × 10^16^
Matrix bond rotation stiffness D_MAT2, N/m^6^	2.39 × 10^22^

## Data Availability

The original contributions presented in this study are included in the article. Further inquiries can be directed to the corresponding authors.

## Abbreviations

The following abbreviations are used in this manuscript:

AI	Artificial intelligence
BBPD	Bond-based peridynamics
CCM	Classical continuum mechanics
CNT	Carbon nanotube
CFRP	Carbon fiber reinforced polymer composite
CLT	Classical laminate theory
DIC	Digital Image Correlation
FE	Finite element
FEM	Finite element model
GFRP	Glass fiber reinforced polymer composite
IRT	Infrared thermography
ML	Machine learning
PD	Peridynamics
PDDO	Peridynamic differential operator
SBPD	State-based peridynamics
SHM	Structural health monitoring
RF_reg	Random Forest Regression
GB_reg	Gradient Boosting Regression
Log_reg	Logistic Regression
THz	Terahertz imaging
